# Sustainable biogenic nanomaterials for green photocatalytic degradation of eosin Y and eosin B in industrial wastewater: a comprehensive review

**DOI:** 10.1039/d6ra03322a

**Published:** 2026-07-02

**Authors:** Komal Shah, Muhammad Zubair, Rida Nisar, Sobia Kunbhar, Gull Sabah Mirza, Mustafa Tuzen, Afzal Shah

**Affiliations:** a National Center of Excellence in Analytical Chemistry, University of Sindh Jamshoro 76080 Pakistan; b Department of Botany, University of Science and Technology Bannu Khyber Pakhtunkhwa 28100 Pakistan; c Department of Horticulture, Faculty of Agriculture Gomal University Dera Ismail Khan Khyber Pakhtunkhwa 29220 Pakistan; d Tokat Gaziosmanpasa University, Faculty of Arts and Sciences, Chemistry Department 60250 Tokat Turkiye mustafa.tuzen@gop.edu.tr; e Department of Botany, University of Punjab Punjab 54590 Pakistan; f Department of Chemistry, Quaid-i-Azam University Islamabad 45320 Pakistan afzal_qau@yahoo.com

## Abstract

Eosin Y and eosin B are persistent organic pollutants extensively used in textile, cosmetic, pharmaceutical, and printing industries, and their discharge into aquatic environments poses serious ecological and health concerns due to their toxicity, stability, and resistance to biodegradation. In recent years, green-synthesized nanomaterials have emerged as promising sustainable photocatalysts for efficient dye degradation under visible and solar-light irradiation. This review comprehensively summarizes recent advances in biogenic photocatalysts derived from plant extracts, microorganisms, and biowaste materials for eosin dye remediation. Particular emphasis is placed on photocatalytic degradation pathways, reactive oxygen species generation, charge-transfer mechanisms, and the role of S-scheme heterojunction-assisted photocatalysis in improving carrier separation and redox efficiency. The effects of operational parameters, including pH, catalyst dosage, irradiation source, and reaction time, are critically discussed alongside the relationships between catalyst structure, morphology, optical behavior, and photocatalytic performance. In addition, conventional and advanced analytical approaches used to investigate degradation pathways, interfacial charge-transfer kinetics, mineralization efficiency, and catalyst stability are highlighted. The review further discusses emerging multifunctional systems integrating dye degradation with photocatalytic hydrogen production and selective chemical synthesis, reflecting the transition from pollutant removal toward solar-driven energy conversion applications. Despite significant laboratory progress, challenges associated with catalyst recovery, stability, light penetration, and industrial-scale implementation remain substantial. Finally, future prospects involving immobilized photocatalysts, piezo-photocatalysis, ferroelectric modulation, coupled catalytic systems, and pilot-scale solar-driven technologies are presented as promising directions for sustainable environmental remediation and energy applications.

## Abstract 2

Eosin Y and eosin B are persistent organic dyes widely used in various industries, whose release into aquatic environments poses significant ecological and health risks due to their toxicity and resistance to biodegradation.

## Graphical abstract

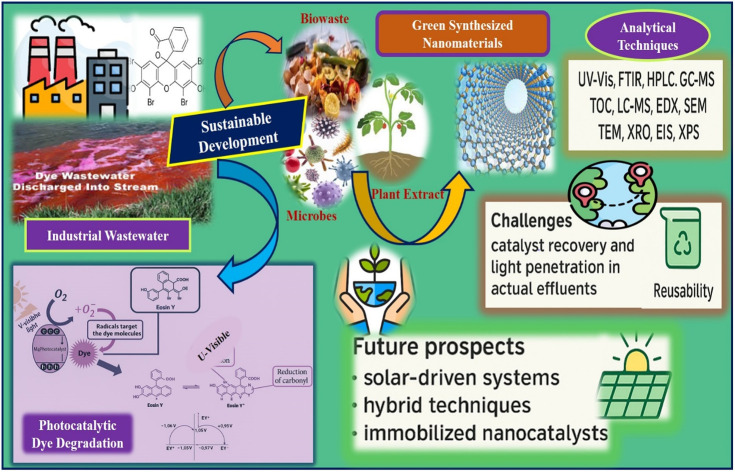

## 1. Introduction

Synthetic dye pollution is a pressing global concern, with eosin dyes including eosin Y (tetrabromofluorescein; Acid Red 87) and eosin B (dibromodinitrofluorescein; Acid Red 91) drawing attention due to their persistence and widespread industrial use. These xanthene-based dyes are favored in textiles, printing, cosmetics, leather, and pharmaceuticals for their vibrant, stable, and cost-effective coloration.^1^ However, there are serious risks to human health and the environment when eosin-containing effluent is improperly disposed of in aquatic environments. Cationic and anionic dyes like eosin can cause skin irritation, carcinogenicity, mutagenicity, and teratogenicity. Therefore, the presence of these dyes in natural and wastewater streams poses significant threats to the aquatic ecosystems, ecological systems and human health.^2^ Notably, the reactive dye-containing effluents discharged from textile industries are responsible for almost 17–20% of global water pollution.^3^ Eosin dyes, being halogenated xanthene compounds, are highly persistent, blocking sunlight, raising Chemical Oxygen Demand (COD), and releasing toxic intermediates that disrupt aquatic ecosystems. Conventional treatments, including coagulation–flocculation, adsorption, biological methods, and even Advanced Oxidation Processes (AOPs), remain largely ineffective for their complete removal.^4^ Conventional chemical treatments are effective but costly and produce secondary pollution. In contrast, green synthesis-based photocatalysis offers a sustainable AOP by using plant extracts or biogenic agents to generate eco-friendly catalysts.^5^

Photocatalysis operates through the excitation of semiconductor materials when exposed to light, producing electron/hole pairs that interact with surrounding water and oxygen. These interactions lead to the formation of reactive oxygen species (ROS), including hydroxyl radicals (OH˙) and superoxide ions (O_2_˙^−^), which are capable of degrading complex dye molecules into simple, non-toxic compounds such as CO_2_ and H_2_O. Among various photocatalysts, TiO_2_ and ZnO are frequently utilized because of their strong oxidation ability, durability, and wide availability. Nevertheless, many traditional synthesis approaches rely on hazardous chemicals, organic solvents, or high-energy conditions, which conflicts with green-chemistry and sustainability principles.

To overcome these challenges, green synthesis has gained attention as a sustainable approach for producing nanomaterials. This method relies on biological sources, such as plant extracts, algae, fungi, and bacteria, as natural reducing and stabilizing agents, avoiding toxic chemicals and energy-intensive processes. This approach not only makes the process environmentally benign but also imparts unique properties to the nanomaterials, such as enhanced surface reactivity and biocompatibility.^6^ Green-synthesized photocatalysts have shown considerable promise in degrading a wide range of organic pollutants, including eosin dyes, under visible or solar light.

Multiple studies have demonstrated that green-synthesized TiO_2_, ZnO, CeO_2_, Fe_3_O_4_, and g-C_3_N_4_ nanoparticles effectively degrade eosin Y and B in both simulated and real wastewater. For example, CeO_2_ nanoparticles synthesized *via C. fistula*, leaf extract achieved over 99% degradation of eosin Y under sunlight within 90 minutes.^7^ Similarly, TiO_2_ nanoparticles derived from *Aloe vera* gel exhibit enhanced photocatalytic activity against eosin B due to the high surface area and improved charge separation. These findings indicate the potential of green nanotechnology to provide eco-friendly and cost-effective solutions for industrial dye pollution.^8^ Despite promising outcomes, the practical application of green photocatalysts faces hurdles such as low recovery, limited reusability, variability in extract composition, and scalability issues. Most studies remain confined to batch-scale lab setups, which often fail to replicate the complexity of real industrial wastewater conditions.^9^ Addressing these limitations requires interdisciplinary research efforts combining material science, environmental engineering, and industrial chemistry.

Analytical characterization techniques are essential for validating photocatalytic performance and understanding degradation mechanisms. Ultraviolet–visible (UV-vis) spectrophotometry, Fourier-transform infrared spectroscopy (FTIR), high-performance liquid chromatography (HPLC), gas chromatography–mass spectrometry (GC-MS), and total organic carbon (TOC) analysis are commonly used to monitor dye decolorization, intermediates, and mineralization. Advanced techniques such as liquid chromatography–mass spectrometry (LC-MS), X-ray photoelectron spectroscopy (XPS), scanning electron microscopy (SEM), transmission electron microscopy (TEM), and X-ray diffraction (XRD) provide structural and compositional insights. Furthermore, photoluminescence (PL) spectroscopy, electrochemical impedance spectroscopy (EIS), and Raman spectroscopy are extensively employed to evaluate charge-transfer dynamics, carrier recombination, and surface stability of photocatalysts.

These techniques collectively enable mechanistic clarity and performance validation. Ultimately, they support scaling green nanomaterials for practical wastewater treatment applications.^10,11^

This review provides a comprehensive and up-to-date analysis of the sustainable photocatalytic degradation of eosin Y and eosin B dyes using green-synthesized nanomaterials, emphasizing their potential for eco-friendly wastewater remediation. It uniquely integrates insights into the chemistry and environmental impacts of eosin dyes with detailed discussions on green synthesis routes employing plant extracts, microorganisms, and biowaste-derived precursors. Comparative evaluation of photocatalytic mechanisms, degradation efficiencies, and operational parameters highlights structure activity relationships across diverse nanomaterials. Moreover, the review identifies existing knowledge gaps and outlines future strategies for scaling up green photocatalytic systems, including immobilized catalysts, solar-driven reactors, and hybrid treatment configurations. This holistic perspective advances understanding toward the practical implementation of sustainable, low-cost, and high-performance photocatalysts in industrial wastewater treatment.

## 2. Eosin dyes: sources, applications, and environmental impact

### 2.1 Types of eosin dyes

The two principal types of eosin dyes are eosin Y (tetrabromofluorescein; Acid Red 87) and eosin B (dibromodinitrofluorescein; Acid Red 91), each distinguished by specific halogenation patterns and characteristic staining properties. Eosin Y is characterized by its bright yellowish-red hue, high water solubility, and strong fluorescence, whereas eosin B contains additional nitro groups, imparting a bluish tint and increased photochemical reactivity.^12^ Eosin dyes exhibit strong visible absorption (*λ*_max_ ≈ 515–530 nm) due to their extended conjugated aromatic systems, supporting uses in staining and microscopy. Halogenation, particularly bromination, enhances their photostability and chemical resistance. While these enhancements are beneficial for industrial uses, they also contribute to the resistance of dyes to biodegradation and oxidative breakdown, leading to environmental persistence.^13^

### 2.2 Industrial applications of eosin dyes

Eosin dyes are widely applied in textiles, where eosin Y is favored for silk, wool, and nylon due to its fiber affinity, photostability, and strong tinting strength. However, low fixation efficiency leads to large dye losses into wastewater. In biomedicine, eosin Y is integral to hematoxylin and eosin (H&E) staining, binding proteins to give cells their diagnostic pink color. It plays a vital role in disease identification through histopathology. Eosin B, with its deeper hue, is used in differential staining to distinguish tissue types.^14,15^ The printing and ink manufacturing sectors also utilize eosin dyes in formulating high-contrast inks for stamp pads, pen inks, and artistic printing. The dyes' excellent solubility in water and alcohols, along with their vivid coloration, makes them ideal for high-visibility printing tasks.^16^ Eosin compounds are used in cosmetics for reddish tints and in diagnostics such as eosin methylene blue (EMB) agar due to their vivid color, stability, and biological compatibility. However, they may cause skin irritation or allergies, and their extensive industrial use leads to significant environmental discharge without proper degradation.^17^

### 2.3 Environmental hazards

Although widely used in industry, eosin dyes represent a significant environmental concern due to their persistent chemical structures, high toxicity, and resistance to conventional treatment processes. Effluents from textiles, printing, and diagnostic laboratories often contain these dyes, which are poorly removed by standard methods such as coagulation and activated sludge. Their stability and complex aromatic frameworks enable them to accumulate in aquatic systems, posing long-term ecological risks.^4^

In aquatic ecosystems, eosin dyes reduce light penetration, inhibiting photosynthesis and disrupting primary production. They also lower dissolved oxygen, impairing aquatic fauna respiration. Studies show acute and chronic toxicity of eosin B to organisms like *Daphnia magna*, zebrafish embryos, and algae. Their harmful effects stem from molecular size, electronegativity, and reactive intermediates formed during photolysis.^18,19^ Eosin dyes pose risks of bioaccumulation by binding to sediments and entering food webs, leading to long-term ecological damage. In humans, occupational exposure can cause dermatitis, respiratory issues, and potential carcinogenic effects. While some regulatory limits exist, environmental release remains poorly controlled, especially in developing regions. Thus, advanced eco-friendly degradation strategies like green nanomaterial-based photocatalysis are urgently needed.^20^

## 3. Principles of photocatalytic degradation of organic dyes

Photocatalytic degradation of organic dyes involves the oxidative breakdown of dye molecules through light-activated semiconductor catalysts, such as TiO_2_, ZnO, or green-synthesized nanomaterials.^21^ Upon light irradiation, electron–hole pairs (e^−^/h^+^) are generated, which subsequently produce reactive oxygen species (ROS), including OH˙ and O_2_˙^−^ radicals. These ROS selectively attack chromophoric structures in dye molecules, particularly aromatic and xanthene rings, through hydroxylation, dealkylation, and ring cleavage, ultimately leading to complete mineralization into CO_2_, H_2_O, and inorganic ions. Photocatalytic efficiency is influenced by catalyst dosage, dye concentration, pH, light intensity, and exposure time, and the use of visible or solar light with green catalysts makes the process sustainable and eco-friendly.^22^

### 3.1 General photocatalytic degradation mechanism

#### 3.1.1 Photon absorption and charge separation

When a semiconductor photocatalyst absorbs photons with energy equal to or greater than its band gap, electrons are excited from the valence band (VB) to the conduction band (CB), leaving positively charged holes in the VB.Semiconductor + *hν* → e^−^ (CB) + h^+^ (VB)

These electron–hole pairs are the primary charge carriers that drive subsequent redox reactions on the catalyst surface.

#### 3.1.2 Generation of reactive oxygen species (ROS)

Photogenerated electrons and holes initiate surface redox reactions, leading to the formation of reactive oxygen species (ROS):

a. Hole oxidation reactions:h^+^ + H_2_O → OḢ + H^+^h^+^ + OH^−^ → OḢb. Electron reduction reactions:e^−^ + O_2_ → O_2_˙^−^c. Subsequent ROS formation reactions:O_2_˙^−^ + H^+^ → HO_2_˙2HO_2_˙ → H_2_O_2_ + O_2_H_2_O_2_˙ + e^−^ → OḢ + OH^−^

#### 3.1.3 Dye degradation

The reactive radicals (OḢ and O_2_˙^−^) attack dye molecules, breaking them down into smaller, non-toxic components:OḢ + dye → degraded intermediates → CO_2_ + H_2_O + mineral acids

**Fig. 1:**
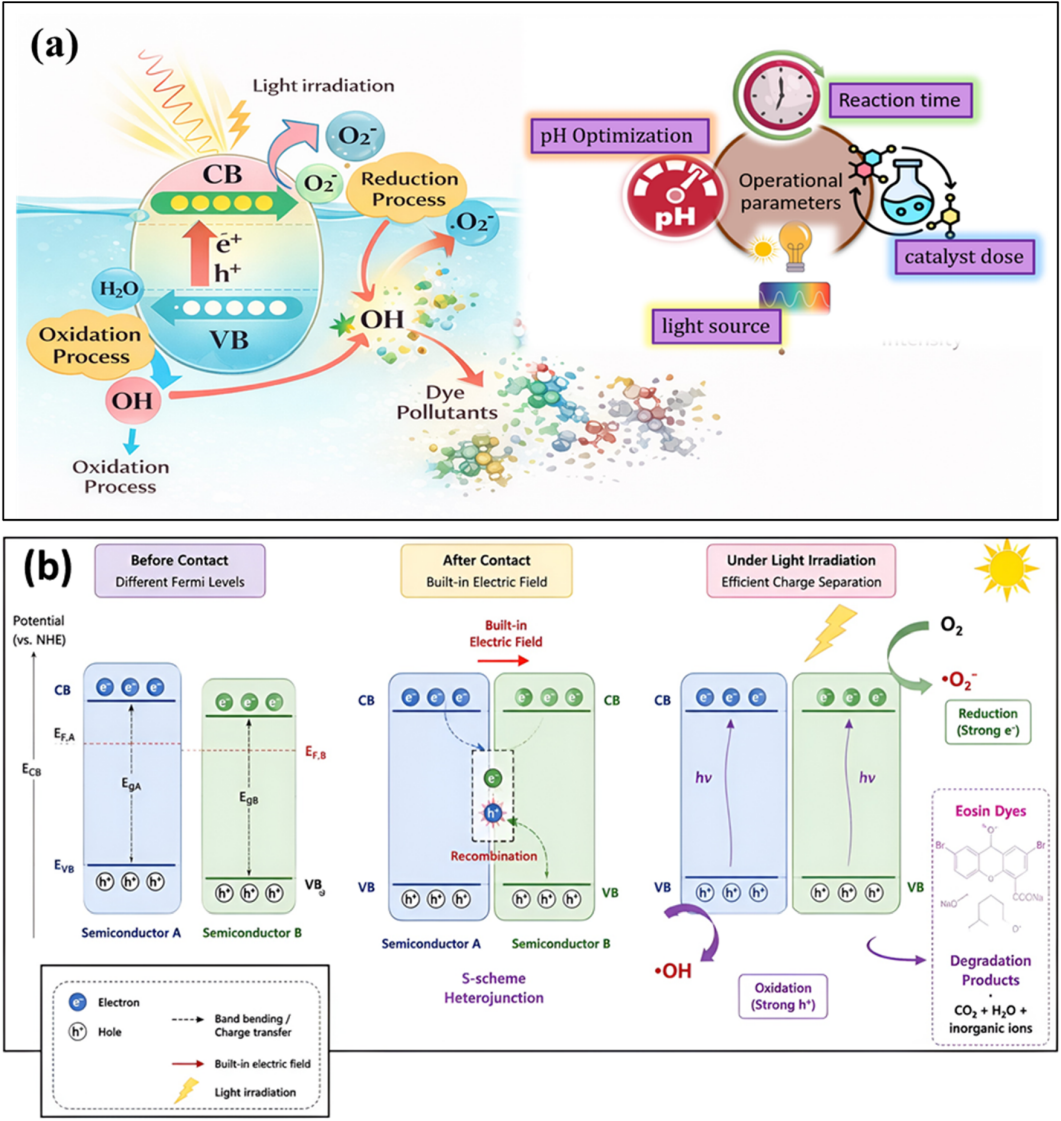
(a) General mechanism of photocatalytic dye degradation in industrial wastewater and operational parameters. (b) Schematic illustration of the S-scheme heterojunction-assisted photocatalytic mechanism.

This process efficiently removes persistent dyes from wastewater using solar or visible light and green catalysts as overviewed in Fig. 1(a).

#### 3.1.4 S-scheme heterojunction-assisted photocatalysis

Recently, S-scheme heterojunction photocatalysts have emerged as an advanced strategy for improving charge carrier separation and photocatalytic efficiency in semiconductor systems. In contrast to conventional type-II heterojunctions, the S-scheme mechanism preserves the strong redox capabilities of photogenerated electrons and holes while minimizing charge recombination through the formation of an internal electric field at the semiconductor interface.^23^ This mechanism has attracted considerable attention for visible-light-driven photocatalytic degradation of organic dye pollutants.^24^

In S-scheme heterojunction (Fig. 1(b)), two semiconductors possessing different Fermi energy levels establish intimate interfacial contact, leading to electron redistribution until thermodynamic equilibrium is attained. This process induces band bending and the formation of a built-in electric field across the heterointerface. Upon light irradiation, photogenerated electrons with lower reduction potential and holes with weaker oxidation potential undergo selective recombination at the interface, whereas highly reductive electrons and strongly oxidative holes are spatially retained in the respective semiconductors. Such a directional charge migration pathway effectively suppresses bulk electron–hole recombination while maintaining superior redox activity for photocatalytic reactions. The enhanced separation and migration of charge carriers significantly promote the formation of reactive oxygen species, particularly hydroxyl radicals (OḢ) and superoxide radicals (O_2_˙^−^), which are principally responsible for oxidative degradation of eosin dyes.^25^ Moreover, S-scheme architectures exhibit improved visible-light harvesting capability, prolonged carrier lifetime, accelerated interfacial electron transfer, and enhanced photocatalytic stability under solar irradiation.^26^

Recent studies have demonstrated that S-scheme g-C_3_N_4_/TiO_2_/CeO_2_ heterostructures exhibit remarkably enhanced photocatalytic performance compared with pristine semiconductors under visible-light irradiation. The optimized photocatalyst achieved degradation efficiency approximately 6.1 times higher than pure g-C_3_N_4_, which was primarily attributed to improved charge separation and accelerated interfacial electron transfer within the S-scheme architecture. XPS and DFT analyses confirmed the formation of strong interfacial interactions among Ti, Ce, and O atoms in the g-C_3_N_4_ framework, facilitating efficient carrier migration and suppressing electron–hole recombination.^27^ Femtosecond transient absorption (fs-TA) spectroscopy helps in elucidating ultrafast charge transfer dynamics, providing direct mechanistic evidence of directional carrier migration and interfacial electron transfer within the heterostructure. This technique resolves real-time excited-state evolution, confirming efficient charge separation at the heterointerface and suppressing recombination losses.^28^ Moreover, the heterojunction significantly enhanced reactive oxygen species generation, particularly OḢ and O_2_˙^−^ radicals, thereby promoting rapid eosin dye degradation. In addition to dye removal, the g-C_3_N_4_/TiO_2_/CeO_2_ S-scheme photocatalyst also demonstrated superior hydrogen evolution activity, prolonged carrier lifetime, enhanced visible-light absorption, and excellent reusability during repeated photocatalytic cycles.^29^ These findings highlight the strong potential of S-scheme heterojunction engineering for developing sustainable multifunctional photocatalysts for wastewater remediation and solar energy conversion applications.^28^

### 3.2 Influence of operational parameters on photocatalytic degradation

Photocatalytic degradation efficiency is highly dependent on operational parameters that regulate ROS generation and pollutant–catalyst interactions. Factors such as pH, light source, catalyst dosage, and reaction time must be optimized to maximize degradation, reduce energy and material use, and ensure environmental safety as depicted in Fig. 1. A clear understanding of these parameters is vital for tailoring photocatalytic systems to specific wastewater conditions and designing reactors for practical applications.^30^

#### 3.2.1 Influence of pH

pH plays a crucial role in eosin dye degradation by altering the catalyst surface charge and dye ionization. In acidic media (pH 3–5), positively charged catalyst surfaces enhance dye adsorption and hydroxyl radical generation, boosting degradation efficiency. However, excessively low pH can compromise catalyst stability, especially in green-synthesized nanomaterials.^31^ Conversely, in alkaline conditions, the catalyst surface acquires a negative charge, leading to repulsion between the catalyst and anionic eosin molecules, thus decreasing adsorption and degradation efficiency.^32^

#### 3.2.2 Influence of light source

The light source in a photocatalytic system provides the energy required to activate the catalyst by driving electrons from VB into CB. The wavelength and intensity of the light are critical factors. Conventional photocatalysts such as TiO_2_ have a wide band gap (∼3.2 eV) and thus require ultraviolet (UV) light for activation. However, UV sources are energy-intensive and less environmentally sustainable.^33^ Green-synthesized or doped nanomaterials with narrowed band gaps enable visible-light activation, reducing energy demands. Harnessing natural sunlight makes photocatalysis cost-effective and sustainable for large-scale wastewater treatment. Thus, aligning catalyst optical properties with the light source is vital for efficient dye degradation.^34^

#### 3.2.3 Influence of catalyst dose

The amount of photocatalyst used in the degradation process is another critical factor. An optimal catalyst dose ensures that sufficient active sites are available for dye molecule adsorption and for photogenerated electron–hole pairs to initiate redox reactions. As catalyst concentration increases, degradation efficiency generally improves due to the availability of more surface area and active sites. However, beyond a certain threshold, excess catalyst loading can cause agglomeration, leading to reduced surface area and increased light scattering, which limits photon penetration. Furthermore, excessive catalyst can promote electron–hole recombination, reducing ROS production and overall photocatalytic activity. Thus, determining the optimal dose is essential to balance performance, material cost, and energy utilization.^35^

#### 3.2.4 Influence of reaction time

The duration of exposure to light directly affects the degradation efficiency of the eosin dye. Longer reaction times generally allow more dye molecules to come into contact with active catalytic sites and more ROS to be generated, promoting complete degradation and mineralization. Photocatalytic degradation often follows pseudo-first-order kinetics, where the rate of reaction is directly proportional to the dye concentration.^36^ The degradation curve typically reaches a plateau as dye molecules are depleted or catalyst sites become saturated. Thus, optimizing reaction time is essential to achieve maximum efficiency while avoiding excess energy use and catalyst fatigue.^37^

## 4. Green and sustainable approaches for photocatalysts synthesis and design

**Fig. 2:**
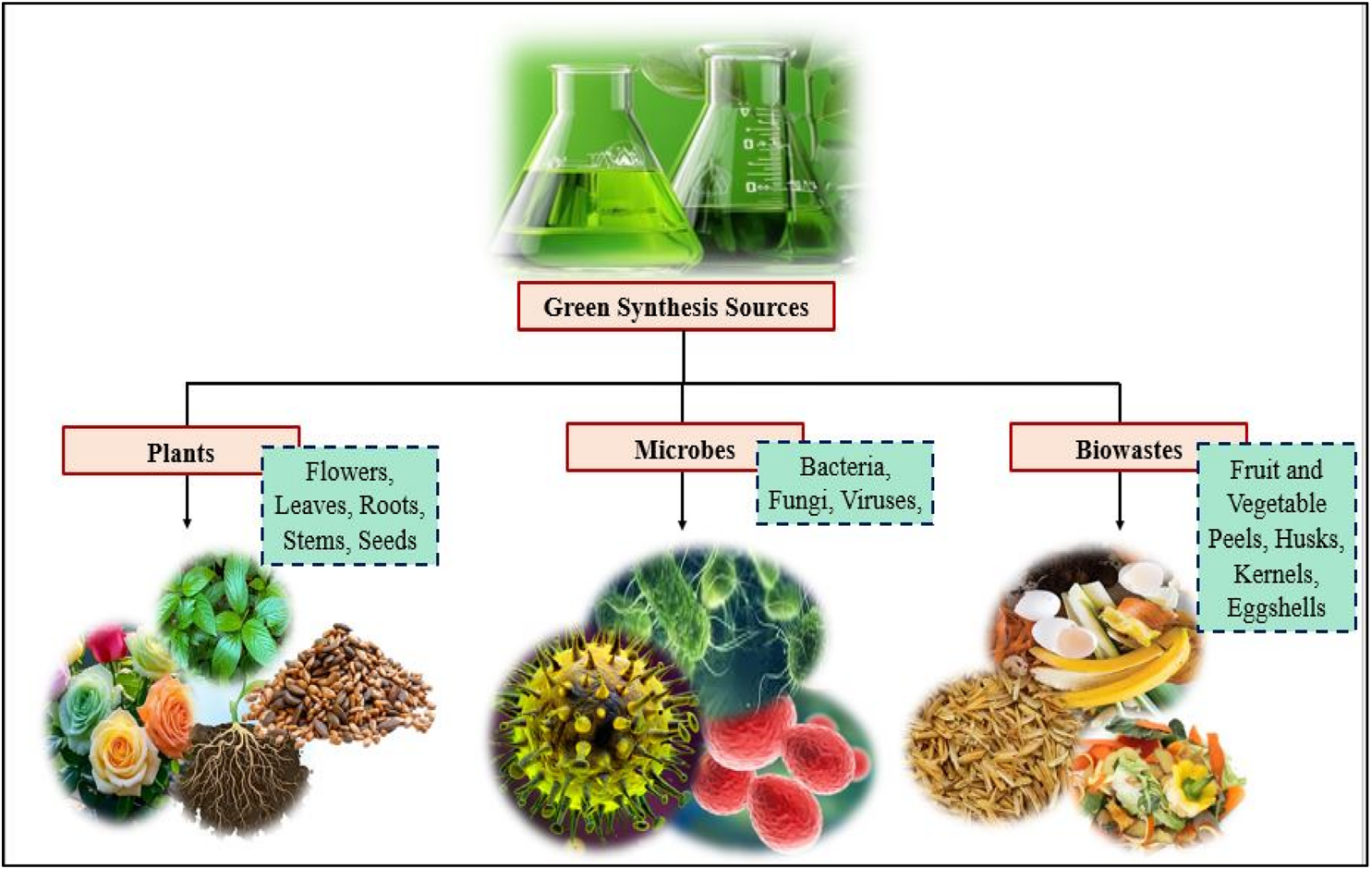
Schematic illustration of the use of green resources, including plant extracts, microbial agents, and biowaste, as eco-friendly and sustainable alternatives for reducing and stabilizing photocatalytic nanomaterials.

**Fig. 3:**
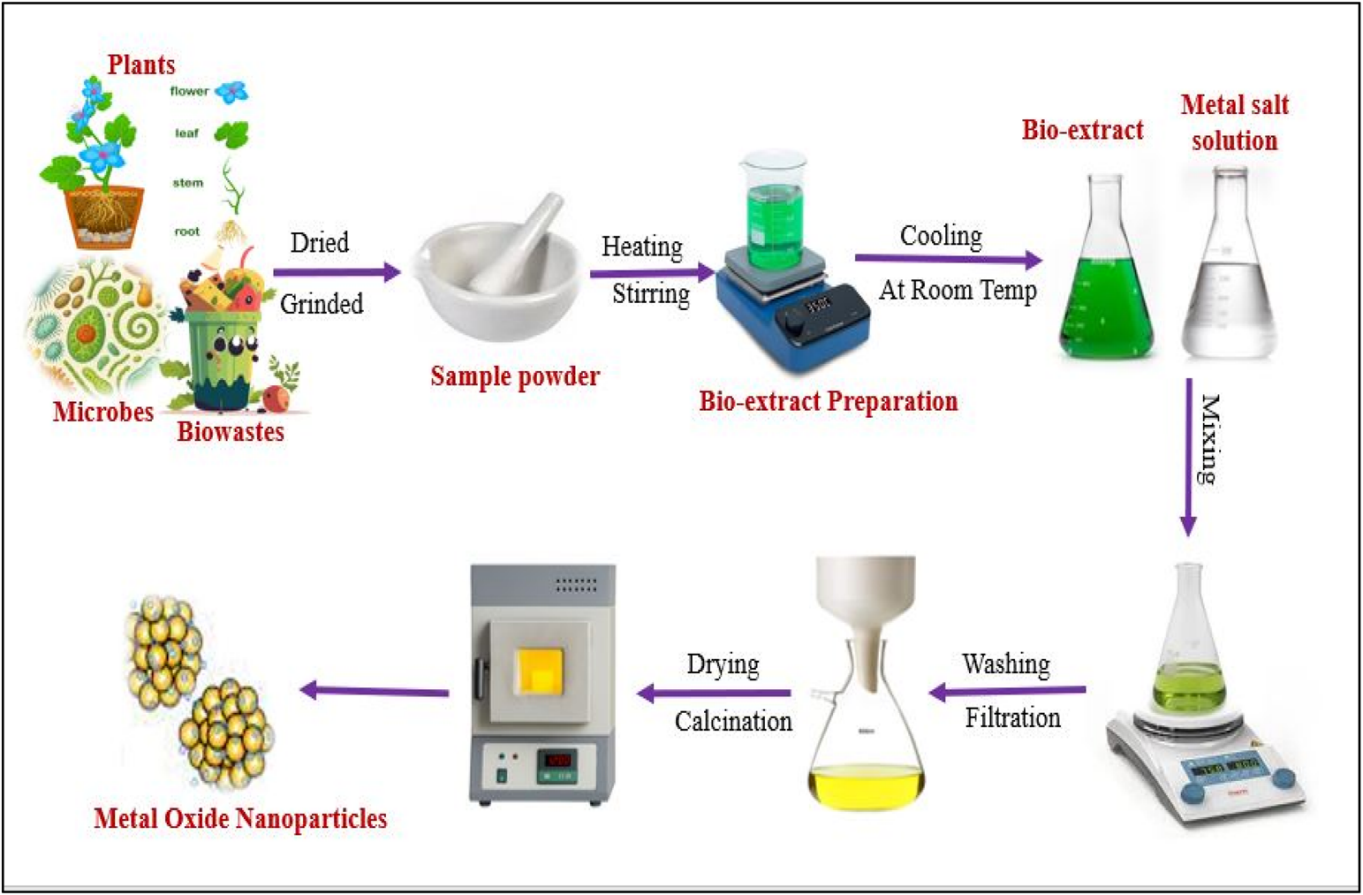
Overview of the green synthesis procedure for nanomaterials.

The pursuit of sustainable technologies has driven interest in green synthesis of photocatalysts as an alternative to conventional, chemical-intensive methods. Traditional synthesis often relies on toxic solvents, harsh reagents, and high-energy inputs, producing hazardous by-products. In contrast, green synthesis employs plant extracts, microbes, and biogenic wastes as natural reducing, stabilizing, and capping agents,^38^ enabling nanoparticle fabrication under mild, eco-friendly conditions, as illustrated in Fig. 2. The procedure begins with the collection of a green source, which may include plant extracts, microbial cultures, or biowaste materials. This is followed by extraction or fermentation methods aimed at isolating bioactive compounds, as illustrated in Fig. 3. These extracts are then mixed with a metal precursor solution, where phytochemicals, enzymes, or biomolecules reduce the metal ions to nanoparticles. Stabilization and capping occur simultaneously, leading to well-dispersed nanoparticles. Finally, the materials are washed, dried, and calcined to obtain the active photocatalyst. This approach lowers environmental toxicity and costs while enabling surface functionalization for enhanced photocatalysis. Green-synthesized ZnO, TiO_2_, CeO_2_, Fe_2_O_3_, and g-C_3_N_4_ have shown strong potential for dye degradation, particularly under visible or solar light.^39^ This section explores the roles of plant-based metabolites, microbial enzymes, and waste-derived bioresources in nanoparticle synthesis, while also highlighting the advantages and real-world relevance of this eco-compatible route.

### 4.1 Plant extracts

Plant-mediated green synthesis is emerging as a key route due to phytochemicals like flavonoids, terpenoids, polyphenols, alkaloids, tannins, and saponins. These compounds act as both reducing and stabilizing agents, enabling metal ion reduction and nanoparticle stabilization under mild, eco-friendly aqueous conditions.^40^ The functional groups (–OH, –COOH, –NH_2_) in the phytochemicals adsorb onto the surface of the growing nanoparticles, limiting agglomeration and controlling particle size and shape.^41^ Various plant species such as *Azadirachta indica* (neem), *Camellia sinensis* (green tea), *Moringa oleifera*, and *Ocimum sanctum* (basil) have been used to synthesize ZnO, TiO_2_, and Fe_2_O_3_ nanoparticles. These green-synthesized photocatalysts often exhibit enhanced photocatalytic activity due to improved crystallinity, surface area, and band gap tuning enabled by the organic residues attached to their surfaces.^42^ Additionally, the use of plant extracts avoids toxic chemicals, aligns with green chemistry principles, and allows for scale-up by utilizing readily available plant species.

### 4.2 Microbes

Microorganisms enable eco-friendly nanoparticle synthesis *via* biomineralization and enzymatic reduction. Bacteria and fungi secrete metabolites like reductase enzymes, amino acids, and polysaccharides that reduce metal ions, with nanoparticles forming either extracellularly in the medium or intracellularly within the cytoplasm.^38^ Bacteria such as *Pseudomonas aeruginosa*, *Bacillus subtilis*, and *Escherichia coli* have been used to synthesize Ag, ZnO, and Fe_2_O_3_ nanoparticles with high surface reactivity. Fungi like *Aspergillus niger* and *Trichoderma harzianum* are also widely studied due to their large biomass, high metal uptake capacity, and ease of cultivation. Algae-based synthesis, though less explored, presents another promising green route with species like *Chlorella vulgaris* showing potential in nanoparticle fabrication.^43^ Microbial-mediated synthesis typically occurs under ambient conditions and offers a high degree of size and shape control. Furthermore, biofunctional coatings from microbial secretions can enhance the stability and photocatalytic efficiency of the resulting nanomaterials, making this approach suitable for environmental applications such as dye degradation.^44^ Microorganisms therefore act as bio-factories where enzymatic reduction, metabolic activity, and biomineralization collectively regulate nanoparticle nucleation, growth kinetics, and surface functionalization, allowing better control over particle uniformity and crystallinity compared to purely chemical routes.^45^

### 4.3 Bio-wastes

Agricultural, food, and household biowastes are low-cost, eco-friendly resources for nanoparticles synthesis. Rich in organic acids, proteins, and minerals, their extracts act as reducing and stabilizing agents, allowing the development of ZnO from orange peel, Fe_2_O_3_ from banana peel, and TiO_2_ from sugarcane bagasse.^46^ The valorization of these bio-wastes reduces landfill burden, promotes circular economy practices, and lowers the cost of nanoparticle production. Moreover, nanoparticles synthesized using waste-derived materials often exhibit superior surface functionalities, which enhance photocatalytic interactions with dye molecules under visible light. Beyond conventional bio-waste precursors, medical waste-derived carbon quantum dots (CQDs) are attracting the attention of investigators for sustainable photocatalytic applications. Recently waste mask-derived CQDs have been utilized in CQD/BiOBr/g-C_3_N_4_ S-scheme heterojunctions for microplastic degradation, where CQDs act as electron mediators to promote interfacial charge separation and directional charge transfer. This strategy provides a sustainable pathway for converting waste into functional photocatalytic materials while supporting circular-economy-based environmental remediation.^47^ In addition to serving as renewable chemical reservoirs, bio-wastes provide heterogeneous organic matrices that facilitate nanoparticle nucleation, enhance surface porosity, and introduce functional groups that improve adsorption and photocatalytic efficiency.^48^

In the last decade, green-synthesized nanomaterials derived from plant extracts, microbes, and bio-waste have shown remarkable efficiency in degrading eosin dyes under solar or visible light. Their eco-friendly synthesis imparts high surface activity, narrow band gaps, and superior photocatalytic performance without relying on toxic chemicals or energy-intensive processes.^49^

Collectively, different biological sources contribute distinct functional roles in green synthesis. Plant extracts mainly provide phytochemical-based reducing and stabilizing agents for rapid nanoparticle formation, while microorganisms enable enzyme-mediated and biomineralization-controlled synthesis with improved structural regulation. In contrast, bio-wastes act as low-cost renewable sources of organic and mineral constituents, supporting both nanoparticle synthesis and sustainable waste valorization. An overview of green-synthesized nanocatalysts found in literature for eosin dye degradation is presented in Table 1. Such comparative analysis not only illustrates the effectiveness of different green routes but also helps identify promising candidates for scale-up and real-world wastewater applications.

**Table 1:** Comparative review of green-synthesized nanomaterials for eosin dye degradation^a^

Nanomaterials	Green source	Synthesis conditions and reagents	Light source	Degradation efficiency (%)	Time (min)	Reference
ZnO NPs	*Mangifera indica*	pH 7; temperature 60–180 °C; time 6 h; reagent: Zn(NO_3_)_2_·6H_2_O	Sunlight	81.2%	120	50
Au-NPs	*Angelica gigas* ribbed stem extracts	pH NR; temperature 80 °C; time 1.5 h; reagent: (AgNO_3_)	UV light	83%	180	51
MgO@BC (ZM)	*Ziziphus mauritiana*	pH: 7; temperature 70 °C; time 5 h; reagent: MgSO_4_·7H_2_O	Uv -visible light	97%	90	52
Ag-NPs	*Camellia japonica* leaf extract	pH:7; temperature 25 °C; time: 1/2 h; reagent AgNO_3_	Sunlight	>97%	60	53
Ag-NPs	*Angelica gigas* ribbed stem extracts	pH NR; temperature 80 °C; time 1.5 h; reagent: HAuCl_4_·3H_2_O	UV light	67%	180	51
MgO@BC (PJ)	*Prosopis juliflora*	pH:7; temperature 70 °C; time 5 h; reagent: MgSO_4_·7H_2_O	Uv visible light	91%	90	52
NiTAPc-SPGO composite	Sugarcane pith	pH NR; temperature 80 °C; time 96 h; reagent: NiPc(NH_2_)_4_	Uv visible light	93.32%	30	54
Ag_2_O–NiO-NPs	(*Citrus sinensis* (L.)*Osbeck*)	pH NR; temperature 70–80 °C; time 1/2 h; reagent: AgNO_3_ and NiCl_2_·6H_2_O	Visible light	65%	<10	55
MgO@BC (AI)	*Azadirachta indica*	pH: 7; temperature 70 °C; time 5 h; reagent: MgSO_4_·7H_2_O	Visible light	92%	90	52
AlONPs	*Carica papaya* seed extract	pH NR; temperature 25 °C; time 1/3 h; reagent: AlCl_3_·6H_2_O	UV light	91.41%	300	56
Ag NPs	*Ophiocoma scolopendrina*	pH:7; temperature 50 °C; time: NR h; reagent: AgNO_3_	Sunlight	96%	15	57
Au-NPs	*Punica granatum* fruit	pH:NR; temperature 25 °C; time: 10–15 min; reagent: AgNO_3_	Sunlight	91%	12	58
Ag NPs	*Trigonella foenum-graecum* seeds	pH:NR; temperature 100 °C; time: 5 min; reagent: AgNO_3_	Sunlight	Upto 100%	6	59
ZnO-NPs	Walnut peel extract	pH: NR; temperature 80 °C; time: 1 h; reagent: ZnCl_2_	Sunlight	95.11%	30	60
Cu/Fe/Ag trimetallic NPs	*C. roseus* leaf extract	pH: NR; temperature 50 °C; time: NR; FeSO4·7H2O, CuSO4·5H_2_O, and AgNO_3_	Sunlight	48.6%	120–360	61
(Fe_2_O_3_–ZrO_2_)	Clove extract	pH: 10.5; temperature 60 °C; time: 1/2 h; reagent: FeCl_3_·6H_2_O and ZrOCl_2_	Sunlight	90%	60–120	32
CNFs	Date palm residues	pH: NR; temperature 280 °C; time: 1 h; reagent: HCL 37%	Sunlight	62.0% to 86.0%	3600	62
CNFs	Date palm residues	pH: NR; temperature 280 °C; time: 1 h; reagent: hydrochloric acid HCL 37%	Sunlight	53.5% to 83.0%	3600	62
Au-Nps	Marine macroalgae	pH: NR; temperature 25 °C; time: 15 min; reagent: AuCl_4_	Sunlight	96%	6	63
CeO_2_ NPs	*Cassia fistula* leaves	pH: NR; temperature 80 °C; time: 2 h; Ce(NO_3_)_3_·6H_2_O	Sunlight	99%	90	7
Au NPs	*Plumeria alba* flower extract	pH:NR; temperature 25 °C; time: 2 min; reagent: gold(iii) chloride trihydrate (HAuCl_4_·3H_2_O)	Visible light	>80%	5	64
AgNPs	*Punica granatum*	pH:NR; temperature 25 °C; time: 10–15 min; reagent: silver nitrate (AgNO_3_)	Solar light	96%	12	58

a Note: NR = not reported in the cited study.

### 4.4 Modulation of intrinsic material properties (ferroelectricity)

In addition to green synthesis routes based on plants, microbes, and bio-wastes, the modulation of intrinsic material properties has emerged as an important strategy to further enhance photocatalytic performance.^65^ Among these, ferroelectricity has gained significant attention due to its ability to generate spontaneous polarization within the crystal lattice. This internal polarization creates built-in electric fields that facilitate the spatial separation and directional migration of photogenerated electron–hole pairs, thereby effectively suppressing recombination losses.^66^ Recent studies have provided clear evidence that ferroelectric polarization plays a crucial role in enhancing photocatalytic charge separation and overall catalytic efficiency. For instance, BiFeO_3_-based heterojunctions coupled with graphitic carbon nitride (g-C_3_N_4_) have demonstrated significantly improved photocatalytic hydrogen evolution and pollutant degradation performance. In particular, Dy-doped Bi_0_._9_Dy_0_._1_FeO_3_ exhibits enhanced ferroelectric polarization, which strengthens the internal electric field and promotes efficient spatial separation of photogenerated electron–hole pairs. Recent work has further demonstrated that ferroelectric polarization in BiFeO_3_/g-C_3_N_4_ Z-scheme heterojunctions directly promotes interfacial charge separation, resulting in markedly improved hydrogen evolution and photocatalytic degradation performance. These findings provide strong experimental validation of ferroelectric polarization as an effective strategy for regulating charge dynamics in photocatalytic systems.^67^ Integrating ferroelectric properties with green-synthesized nanomaterials provides a promising synergistic pathway, combining sustainable fabrication with advanced electronic structure engineering to achieve high-performance photocatalysts for environmental remediation applications.^66,68^

### 4.5 Mechanistic differences between bio-based and conventional nanomaterials in photocatalysis

Bio-based and non-bio-based nanomaterials exhibit fundamentally different behaviors in photocatalytic degradation reactions due to variations in surface chemistry, defect density, and interfacial charge transfer dynamics.^69^ Bio-based nanomaterials, derived from plant extracts, microbes, or bio-wastes, typically possess surface-bound organic functional groups, heteroatom incorporation, and oxygen vacancies introduced during green synthesis.^70,71^ These features enhance pollutant adsorption, facilitate electron trapping, and promote efficient charge separation, ultimately accelerating the generation of reactive oxygen species such as OH˙ and O_2_˙^−^.^71^ In contrast, non-bio-based nanomaterials synthesized *via* conventional chemical routes generally exhibit cleaner and more crystalline surfaces with fewer intrinsic defects, resulting in weaker adsorption capacity and reduced interfacial interaction with target pollutants. As a result, they often require external doping, surface modification, or co-catalysts to achieve comparable photocatalytic activity.^72,73^ Mechanistically, bio-based systems favor adsorption-assisted degradation pathways, whereas conventional systems rely predominantly on band structure-driven redox processes, leading to differences in reaction kinetics and overall degradation efficiency^74^

Conclusively, green synthesis of photocatalysts using plant extracts, microbes, and bio-wastes offers a sustainable and eco-friendly alternative to conventional methods, enabling efficient nanoparticle formation with enhanced surface and optical properties. These approaches significantly improve photocatalytic performance for environmental applications such as dye degradation. In addition, recent advances highlight ferroelectricity as an important intrinsic property, where internal polarization fields enhance charge carrier separation and suppress recombination. The combination of green synthesis strategies with intrinsic material property engineering provides a promising pathway for the development of efficient and sustainable photocatalysts.

## 5. Mechanisms and kinetics

### 5.1 Eosin degradation pathway (intermediates and products)

**Fig. 4:**
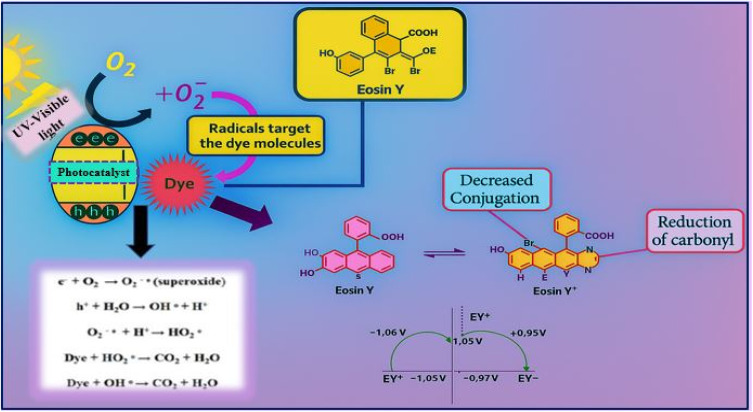
Mechanism of the photolytic biodegradation of eosin dye into harmless compounds.

Photocatalytic degradation of eosin dyes is driven by ROS generated under light irradiation with semiconductor nanomaterials. Light excites electrons from the VB to CB, creating electron–hole pairs that initiate redox reactions. The resulting OH˙ and O_2_˙^−^ radicals oxidatively attack the dye's chromophoric structures, particularly xanthene and aromatic rings, causing stepwise molecular cleavage and degradation^75^ as described in Fig. 4, respectively.

The photocatalytic degradation of eosin dyes typically follows a sequential pathway beginning with de-ethylation and de-bromination, which destabilize the dye structure. Subsequent aromatic ring opening generates intermediates like phthalic acid, benzoquinone, maleic acid, and formic acid. These intermediates are oxidized into simpler organic acids and finally mineralized into CO_2_, H_2_O, and inorganic ions (Br^−^, NO_3_^−^). The efficiency of this process is governed by catalyst properties, light intensity, and reaction conditions, while analytical tools such as LC-MS, GC-MS, and FTIR confirm intermediate formation and complete mineralization.^10,76^

### 5.2 Langmuir–Hinshelwood kinetics

The Langmuir–Hinshelwood (L–H) kinetic model is used to define the photocatalytic degradation behavior of organic pollutants, including eosin dyes, on the surface of heterogeneous catalysts such as green-synthesized nanomaterials. This model accounts for two essential processes (1) the adsorption of reactant molecules (*e.g.*, dye): onto the catalyst surface, and (2) their subsequent reaction with active species (*e.g.*, OH˙, h^+^, O_2_˙^−^) generated by light-activated photocatalysts. It is based on the assumption that the surface of the photocatalyst offers a finite number of active sites, and that the reaction rate is proportional to the surface coverage of the adsorbed dye molecules.^77,78^ Mathematically, the general L–H rate expression is:
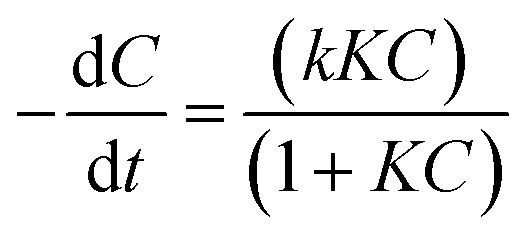
where:

• *C* = concentration of the dye (*e.g.*, eosin) at time *t* (mg L^−1^)

• d*C*/d*t* = rate of degradation (mg L^−1^ min^−1^)

• *k* = intrinsic reaction rate constant (mg L^−1^ min^−1^)

• *K* = Langmuir adsorption equilibrium constant (L mg^−1^)

This expression reflects a non-linear relationship between the degradation rate and dye concentration, accounting for both adsorption dynamics and surface-mediated reactions. Under dilute dye concentrations (KC ≪ 1), the Langmuir–Hinshelwood model simplifies to pseudo-first-order kinetics, where the apparent rate constant is given by:*k*_app_ = *kK*

Therefore,
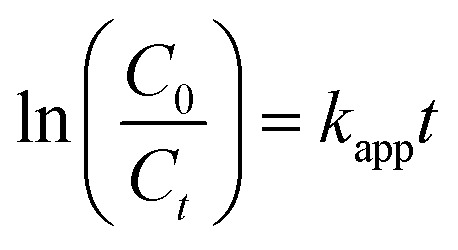
where:

• *C*_0_ and *C*_*t*_ are the concentrations at initial and time *t*.

• *k*_app_ is the apparent pseudo-first-order rate constant (min^−1^).

This linear form is frequently used to calculate degradation rate constants from experimental results *via* the plot of ln(*C*_0_/*C*_*t*_) *versus* time. A straight-line fit indicates the reaction follows pseudo-first-order kinetics under the tested conditions.

Nevertheless, at higher dye concentrations, active sites become saturated, and the model transitions toward zero-order behavior, where the rate becomes independent of dye concentration. This deviation indicates a mass transfer limitation, a saturation of reactive species (*e.g.*, hydroxyl radicals), or a significant drop in light penetration due to excessive dye shielding (inner filter effect). In such cases, it becomes necessary to consider multi-site adsorption, competitive interactions, and diffusion-controlled kinetics, especially in real wastewater conditions where multiple solutes are present.^78,79^

The values of the intrinsic reaction rate constant (*k*) and the adsorption equilibrium constant (*K*) in the Langmuir–Hinshelwood model are highly dependent on experimental conditions such as:

• Catalyst loading: more active surface area enhances adsorption, but excessive loading can cause light scattering.

• Initial dye concentration: determines the level of surface site occupation and reaction saturation.

• Light intensity and wavelength: directly affect ROS generation and catalyst excitation.

• pH and temperature: influence both dye ionization and catalyst surface charge.

Several reports have examined eosin removal using green-synthesized photocatalysts. Incorporating Gd and g-C_3_N_4_ into ZnO significantly boosted the activity of pristine ZnO, increasing degradation efficiencies from roughly 20% to 96% and raising the apparent rate constant from about 1.18 × 10^−3^ to 19.23 × 10^−3^ min^−1^. The photodegradation process conformed to the Langmuir–Hinshelwood mechanism and showed pseudo-first-order kinetics. The improved photocatalytic response of the modified ZnO systems was mainly due to the synergistic influence of Gd and g-C_3_N_4_, which enhanced light utilization and facilitated more effective generation and separation of charge carriers.^50,80^ Overall, the Langmuir–Hinshelwood model provides a robust theoretical and practical framework for understanding and optimizing photocatalytic dye degradation, as overviewed in Fig. 4, which is crucial for scaling up photocatalytic systems for industrial effluent treatment.^60,80^

### 5.3 Photocatalyst reusability and stability

Reusability and long-term stability are crucial for the practical usage of green-synthesized photocatalysts. While their surface reactivity enhances degradation efficiency, it also increases risks of deactivation, agglomeration, and leaching over repeated cycles. Reusability is assessed through cyclic degradation tests, where photocatalysts are recovered by centrifugation, filtration, or magnetic separation and reused under similar conditions. Efficiency loss typically arises from surface fouling, aggregation, or intermediate adsorption. Notably, green-synthesized ZnO nanoparticles showed only a 6–10% decline in eosin degradation after 4–5 cycles, underscoring the value of effective catalyst regeneration.^81^ Stability refers to maintaining crystallinity, morphology, and oxidation states of photocatalysts during repeated use and light exposure. Metal oxides like ZnO, TiO_2_, and CuO risk photo-corrosion, while organic residues in green-synthesized catalysts may degrade or desorb over time. Techniques such as XRD, FTIR, SEM, and EDS are commonly used to assess structural integrity before and after degradation.^50,82,83^

The following approaches have been explored to mitigate degradation and enhance reusability:

• Surface functionalization using bio-compatible capping agents or polymers to reduce nanoparticle agglomeration.

• Composite formation with inert or porous supports (biochar, or clay) to enhance stability and prevent leaching.^52^

• Magnetization using Fe_3_O_4_ cores or shells for easy magnetic separation and recovery from treated solutions.^84^

A consolidated summary of the photocatalyst reusability and degradation efficiency over cycles is presented in Table 2, which outlines structural and performance retention across various green-synthesized systems.

**Table 2:** Reusability and degradation efficiency of various green-synthesized photocatalysts against eosin dye

Photocatalyst	Number of cycles	Efficiency retention (%)	Remarks	Reference
Co_3_O_4_ Nps	4	94.2%	No significant loss of activity was observed after four cycles	76
Cit-Fe_3_O_4_@TiO_2_ nanocomposite	5	96%	Reusability results highly suggest the use of Cit-Fe_3_O_4_@TiO_2_ magnetic nanoparticles as a prospective catalyst for the organic dyes	85
TiO_2_@ITO nanocomposite	5	99.8%	Suggested an exceptional icon at the commercial level for the degradation of pollutants	86
Ag–Fe_3_O_4_ NPs	3	90.12%	Efficient photocatalyst for the removal of harmful dyes from industrial wastewater	84
CNFs	5	81%	The findings reveal the adsorbate molecules' maximal adsorption capability for eosin Y	62
Au(Salen)@CC composite	4	96%	Capable of new multifunctional applications	87
Fe_2_O_3_–ZrO_2_	5	74%	The present adsorbent remains effective for only a few cycles	32
Ni/ZnO NPs	4	90.7%	The reusability of the produced nanocatalyst confirms its stability and efficiency over multiple cycles	88
MgO@BC (ZM)	5	97%	Remarkable degradation efficiency for wastewater treatment	52
CeO_2_@rGO nanocomposites	3	99.6%	High-performance photocatalysts for wastewater treatment applications	75
Cr, N Co-doped TiO_2_ NPs	3	88%	Efficiently removed organic pollutants	82
CuO NFs	5	86%	The engineered catalyst could be used at the commercial level	83

## 6. Analytical methods used in eosin dye degradation

The effective assessment of photocatalytic degradation of eosin dyes relies heavily on analytical methods that allow researchers to monitor reaction progress, identify intermediates, confirm mineralization, and evaluate catalyst reusability. These tools are integral for validating mechanisms and optimizing degradation efficiency. The following section provides an in-depth overview of conventional and advanced analytical techniques applied in recent scientific literature up to 2025.

### 6.1 UV-visible spectrophotometry

UV-visible spectrophotometry is the most commonly employed technique to monitor the photocatalytic degradation of eosin dyes due to its simplicity, speed, and affordability. It is based on Beer–Lambert's Law, which correlates the absorbance of light to the concentration of the absorbing species. Eosin dyes, particularly eosin Y and eosin B, exhibit strong absorbance in the visible region around 514–530 nm, associated with their xanthene chromophore. During degradation, a progressive decline in absorbance intensity at this *λ*_max_ indicates chromophore cleavage and dye decolorization.^81^

Table 3 shows that applied UV-vis spectroscopy to monitor Eosin B degradation catalyzed by aluminium oxide nanoparticles (AlONps) under visible light. The authors correlated the reduction in absorbance to increasing Ag content, concluding enhanced surface plasmon resonance effects promoted dye breakdown. Despite its utility, UV-vis spectrophotometry cannot detect intermediates or confirm complete mineralization. Thus, it is often complemented by TOC, HPLC, or GC-MS to fully characterize the degradation process.^56^

**Table 3:** Summary of conventional and advanced analytical tools used to investigate eosin dye photocatalytic degradation

Photocatalyst	Objectives	Analytical technique	Outcomes	References
AlO-Nps	Nps formation	UV-vis spectrophotometry	Absorption peak at a wavelength of 272 nm was detected	56
Ag-NPs	Monitor Nps formation	UV-vis spectrophotometry	Sharp peak of synthesized nanoparticles was detected	53
Ag-NPs	Surface bond change	FTIR spectroscopy	Observed functionalities shows successful oxidation of Nps	53
Cr, N Co-doped TiO_2_ Nps	Intermediate separation	HPLC	Possible degradation pathways were made for both dyes	82
AlO-Nps	Identify unknown volatile products	HPLC	Prominent peaks were identified as the presence of catechin and quercetin	56
Ag-Nps	Detect volatiles chemical constituents	GC-MS	Sphere-like Ag-NPs exhibit excellent degradation of EY dye	53
AlO-Nps	Determine degradation extent	GC-MS	Results recognized the presence of five prominent bioactive compounds	56
CeO_2_@rGO nanocomposites	Assess mineralization	TOC analysis	The results highlight the potential of nanocomposites as high-performance photocatalysts for wastewater treatment	75
ZnO	Measure TOC drop	TOC analysis	The results show a significant mineralisation of EY dye, which drops the formation of potentially harmful agents	89
CuFe_2_O_4_	Compare mineralization level	Raman spectroscopy	Efficiently remove organic pollutants	82
TiO_2_	Structural, and photocatalytic properties	Raman spectroscopy	The presence of TiO_2_ co-doped with nickel (Ni) and indium (In) in the coatings was confirmed	90
NiTAPc-SPGO nanocomposite	Charge transport	EIS	Photocatalysts can enhance the separation and transfer efficiency of the photogenerated e^−^/h^+^ pairs, thereby improving the efficiency of photocatalytic reactions	54
AgNps	Catalyst efficiency evaluation	EIS	EIS plot generally shows lower electron transfer resistance, which usually leads to quick charge shift and more successful segregation	91
CeO_2_ NPs	Recombination insight	PL spectroscopy	Applied for the photocatalytic breakdown of EY under direct sunlight	7
AuNPs	Product identification	LC-MS	The degradation intermediates identified	77
Cr, N Co-doped TiO_2_ Nps	Advanced pathway tracing	LC-MS	Possible degradation pathways were made for both dyes	82
(EG)–ZnO nanocomposite	Surface chemistry analysis	XPS	The photoelectrochemical degradation process resulted in enhanced degradation efficiency	92
ZnO/ZnS	Structure and composition of the composites	XPS	The resulting photocatalyst has good degradation effect on eosin	93
CuO NFs	Topography, crystallinity	SEM	Nanoflakes like morphology of PVP-CuO was confirmed while the average size of the prepared material was calculated 31.8 nm	83
(AgNPs@Ac)	To analyze the surface morphology	SEM	Nanoparticle was found to be spherical in shape	94
SNPs	Particle size range and stability	TEM	Spherical morphologies with homogeneous particle size distribution have been observed	91
(AgNPs@Ac)	Particle size range and stability	TEM	Nanoparticle was found to be spherical in shape, having particle size value ranged from 50 to 100 nm	94
Cit-Fe_3_O_4_@TiO_2_	Elemental composition	EDX	Titania covering particle size was changed to 15.8 nm due to the coating TiO_2_ shell over Fe_3_O_4_	85
Ni/ZnO Nps	Elemental composition	EDX	Results suggested that a Ni/ZnO nanocatalyst could be a potential nanomaterial for developing an enhanced doped nanocatalyst for EB removal	88
Ag-Nps	Crystalline pattern	XRD	Excellent catalyst for the reduction of hazardous and toxic dyes	94
SNps	Crystalline pattern	XRD	Promising and sustainable materials for photolytical dye degradation	91

### 6.2 Fourier transform infrared spectroscopy

FTIR spectroscopy plays a critical role in characterizing chemical changes that occur during the photocatalytic degradation of eosin dyes. Based on the principle of infrared absorption due to molecular vibrations, FTIR detects functional group transformations, such as the breakdown of dye chromophores and the formation of oxidative products. The technique is particularly valuable for identifying the cleavage of aromatic rings, halogen substituents (*e.g.*, –Br), nitro groups, and the emergence of oxygenated moieties such as –OH, –COOH, or carbonyls (C=O).^6,53^

Karthik *et al.* investigated eosin B degradation using AgO nanoparticles synthesized with Aloe vera extract. FTIR spectra revealed the disappearance of bands around 1600 cm^−1^ corresponding to aromatic C=C stretching and peaks near 500 cm^−1^ related to C–Br, confirming effective chromophore breakdown and dehalogenation. Concurrently, new peaks near 3400 cm^−1^ and 1720 cm^−1^ indicated hydroxyl and carbonyl group formation, implying oxidative degradation pathways driven by reactive oxygen species.^53^ Despite being qualitative, FTIR is vital for monitoring molecular changes in photocatalysis. Coupled with UV-vis, HPLC, and TOC, it enables a comprehensive understanding of dye degradation in green systems.^5^

### 6.3 High-performance liquid chromatography (HPLC)

HPLC is a highly sensitive and selective analytical technique used to separate, identify, and quantify intermediates and final degradation products formed during the photocatalytic treatment of dyes like eosin Y and eosin B. The method works based on the differential interaction of analytes with the stationary phase and their affinity to the mobile phase, thus allowing separation by polarity and molecular weight under high pressure. It plays a crucial role in mechanistic pathway elucidation, confirming whether degradation involves complete mineralization or merely transformation into other organic compounds.^5,56^

In addition, HPLC–MS was integrated with traditional HPLC to confirm structures of trace-level intermediates during eosin B degradation with TiO_2_ oxide nanocomposites.^82^ This combination allowed them to trace minor aromatic intermediates that may contribute to long-term toxicity if not fully mineralized, as listed in Table 3. HPLC thus remains a cornerstone analytical method in validating photocatalytic degradation efficiency, providing quantitative and qualitative data on dye removal, intermediate fate, and potential environmental impacts.

### 6.4 Gas chromatography-mass spectrometry (GC-MS)

GC-MS is a highly sensitive and informative analytical technique used for the detection, separation, and structural elucidation of volatile and semi-volatile organic degradation products resulting from the photocatalytic breakdown of dyes like eosin Y and eosin B. The GC part separates the individual components of a complex mixture based on boiling point and volatility, while the mass spectrometry (MS) identifies the molecular structure of each compound by analyzing its mass-to-charge (*m*/*z*) ratio. This dual system makes GC-MS exceptionally useful for exploring oxidative degradation pathways and determining the extent of dye mineralization.^53^

In a recent study,^56^ used GC-MS to study eosin B degradation under solar-assisted photocatalysis using AlO nanoparticles. The method enabled the identification of intermediate compounds such as muconic acid, fumaric acid, and malonic acid, suggesting that the dye underwent oxidative ring opening followed by successive decarboxylation. The degradation profile helped to reconstruct a plausible mechanistic pathway, reinforcing the effectiveness of the photocatalyst, as described in Table 3. The integration of these methods provided a more complete view of photocatalytic efficiency beyond just decolorization. In summary, GC-MS stands as a cornerstone analytical method for confirming the fate of eosin dye molecules during and after degradation. It is particularly useful when combined with HPLC, TOC, and FTIR for comprehensive evaluation of photocatalytic systems, especially in applications targeting green, sustainable, and complete mineralization routes.^79^

### 6.5 Total organic carbon (TOC) analysis

TOC analysis is a quantitative technique used to determine the amount of carbon found in organic compounds present in a sample. In photocatalytic degradation studies, TOC serves as a direct and reliable indicator of mineralization, representing the degree to which a dye has been broken down into inorganic end-products such as CO_2_ and H_2_O. Unlike UV-Visible spectroscopy or HPLC, which focus on chromophore decolorization or detection of intermediates, TOC provides a holistic measurement of the total organic content remaining post-treatment.^95^

In a recent study, Gayathri *et al.* utilized TOC analysis to evaluate the mineralization efficiency of CeO_2_@rGO nanocomposites synthesized *via* a green method. Under solar irradiation, they observed up to 90% reduction in TOC after 60 minutes, which closely correlated with >90% decolorization detected *via* UV-vis analysis. The combination of high TOC removal and absorbance reduction confirmed both visible dye removal and chemical mineralization, as mentioned in Table 3. Moreover, TOC analysis is often used in conjunction with GC-MS or HPLC to quantify the persistence of intermediates and identify any resistant organic fractions.^75^ Its ability to measure total carbon content irrespective of molecular structure makes it an indispensable part of modern wastewater treatment research and validation.^95^

### 6.6 Electrochemical impedance spectroscopy (EIS)

EIS is a powerful diagnostic tool used to investigate the electrical properties and charge-transfer behavior of photocatalytic systems at the electrode–electrolyte interface. It measures the system's impedance in response to an applied alternating current (AC) over a range of frequencies, providing insight into electron mobility, recombination rates, interfacial resistance, and overall catalytic efficiency. A lower charge transfer resistance (*R*_ct_) indicates improved separation and transport of photoinduced electron–hole pairs, crucial for the photocatalytic degradation of dyes such as eosin Y and eosin B.^7,54^

Afzal Shah and coworkers studied green-synthesized CeO_2_ nanoparticles for eosin B degradation, employing EIS to monitor variations in reaction during repeated photocatalytic cycles.^7^ The results indicated a gradual increase in impedance over cycles, correlating with a decline in degradation efficiency, highlighting EIS as a sensitive indicator of catalyst aging and stability. Its ability to correlate electrical resistance with photocatalytic performance makes it an indispensable tool for developing next-generation green nanomaterials for eosin dye degradation,^7^ as seen from the previous literature presented in Table 3.

### 6.7 Photoluminescence (PL) spectroscopy

PL spectroscopy is a widely used optical technique for evaluating the recombination behavior of photogenerated charge carriers (electrons and holes) in semiconductor photocatalysts. When a photocatalyst absorbs light, electrons are excited to CB, leaving holes in VB. If these carriers recombine rapidly, the photocatalytic efficiency drops. PL spectroscopy detects this radiative recombination by measuring the light emitted as electrons fall back to VB. A lower PL intensity indicates reduced recombination rates, implying more efficient charge separation, which directly correlates with enhanced photocatalytic performance.^56^

Saha *et al.* studied bio-derived AlONps nanoparticles and observed diminished PL intensity when synthesized using bio extract. The authors attributed this to the presence of phytochemicals, which enhanced surface defect states that promoted charge trapping and transfer, ultimately improving photocatalytic activity for eosin dye removal, as listed in Table 3. In addition to assessing recombination rates, PL spectroscopy also provides insight into trap states, oxygen vacancies, and surface defects, all of which influence photocatalytic reactivity. This makes PL an invaluable tool not only for screening catalyst quality but also for guiding the rational design of photocatalytic materials.^56^ When integrated with electrochemical techniques such as EIS and structural analyses like XRD or SEM, it offers a holistic understanding of material efficiency in degrading persistent dyes like eosin Y and B under visible or solar light.^91^

### 6.8 Liquid chromatography–mass spectrometry (LC-MS)

LC-MS is a hyphenated analytical technique that integrates the separation capabilities of HPLC with the molecular specificity of Mass Spectrometry (MS). This combination is particularly powerful for analyzing non-volatile, thermally labile, and polar degradation products, which cannot be easily detected using GC-MS. LC-MS is thus considered one of the most advanced and informative tools for elucidating photocatalytic degradation pathways in aqueous media.^82^

In a recent study, Nagaraja *et al.*^77^ applied LC-MS to monitor eosin B degradation in real wastewater treated with AuNPs-based photocatalysts. The analysis revealed transient formation of aromatic carboxylic acids and amine derivatives, offering mechanistic clarity on deamination, demethylation, and dehalogenation reactions under visible light. The ability of LC-MS to detect molecular ions with high accuracy made it instrumental in mapping the degradation route, as demonstrated in Table 3. While HPLC provided retention-based separation, LC-MS further identified molecular structures and fragmentation patterns, giving a much deeper understanding of the underlying chemical transformations. Its role is indispensable in studies aiming to understand the complete fate of dye molecules and to evaluate the environmental safety of intermediate and final products generated during photocatalytic treatment.^9,82^

### 6.9 X-ray photoelectron spectroscopy (XPS)

XPS is a powerful surface-sensitive analytical technique used to investigate the elemental composition, oxidation states, and chemical environment of photocatalysts before and after their use in dye degradation. It operates on the principle of the photoelectric effect, where X-ray photons bombard a material's surface and eject core-level electrons. The binding energies of these photoelectrons are characteristic of specific elements and their oxidation states, allowing researchers to deduce redox behavior, surface chemical modifications, and active sites on the catalyst surface.^90^

In a recent study,^75^ CeO_2_@rGO nanocomposites were synthesized to examine the valence states of Ce and O in the samples that contained carbonaceous adsorbents after photocatalytic activity, correlating with enhanced eosin B degradation. The results clearly show the main components of Ce 3d, O 1s, and C 1s. Ce^4+^ 3d_3/2_ were identified as expected and clearly shown from the literature presented in Table 3. Furthermore, XPS is crucial for evaluating catalyst reusability and stability, as binding energy shifts or surface oxidation in long-term studies indicate deactivation. By tracking oxidation states, surface composition, and redox interactions, it guides the design of durable green photocatalysts for persistent dyes like eosin.^90^

### 6.10 Energy dispersive X-ray spectroscopy (EDX)

EDX is an essential technique used in conjunction with electron microscopy (typically SEM or TEM) to determine the elemental composition of photocatalysts. When a high-energy electron beam interacts with a sample, it generates characteristic X-rays from the constituent elements. EDX detects these X-rays and provides qualitative and semi-quantitative analysis of the elements present. In studies involving the degradation of eosin Y and eosin B dyes, EDX is employed to confirm the presence of expected elements (Ti, Zn, Ni, O) in green-synthesized nanomaterials and to detect any impurities or dopants introduced during synthesis. Additionally, EDX mapping allows visualization of the spatial distribution of elements across the photocatalyst surface. This information is vital for correlating elemental uniformity and composition with photocatalytic performance and stability.^85,88^

### 6.11 Raman spectroscopy

Raman spectroscopy is a non-destructive vibrational spectroscopic technique used to investigate molecular vibrations, crystal structures, and bonding characteristics in materials. It operates based on the inelastic scattering of monochromatic light, which interacts with phonons or molecular vibrations in the material. The resulting Raman shifts provide fingerprint information that is highly specific to lattice structures, bond types, and molecular symmetry. In photocatalytic degradation studies, Raman spectroscopy is vital for assessing crystallinity and structural stability of catalysts before and after use. Its sensitivity to metal–oxygen lattice vibrations makes it ideal for confirming phase integrity and detecting transitions induced by stress or reuse.^96^ Previously conducted research,^82^ complemented their XRD and FTIR analysis with Raman to confirm the presence of oxygen vacancies in TiO_2_, a key factor promoting ROS generation during eosin dye degradation. The emergence of defect-related bands in the Raman spectra aligned well with improved photocatalytic performance, as demonstrated in Table 3 Raman spectroscopy is crucial for assessing phase stability, detecting lattice defects, and validating structural transitions in catalysts during reuse and long-term operation.^96^

### 6.12 Scanning electron microscopy (SEM)

SEM is a widely used analytical technique to investigate the surface morphology and microstructural features of photocatalysts employed in dye degradation. SEM provides high-resolution images by scanning a focused electron beam over the sample surface, enabling visualization of particle shape, size, and surface texture. In the context of eosin dye degradation, SEM analysis helps assess the uniformity of green-synthesized nanomaterials, identify agglomeration or porosity, and monitor morphological changes before and after photocatalytic cycles. These insights are crucial for understanding how surface properties influence the adsorption and photocatalytic behavior of nanocatalysts.^54,82,97^

### 6.13 Transmission electron microscopy (TEM)

TEM offers a deeper look into the internal structure and nanoscale features of photocatalysts. By transmitting electrons through an ultra-thin specimen, TEM allows for high-resolution imaging of particle size, shape, and crystal lattice arrangements. It is especially useful for characterizing the fine structure of green-synthesized nanomaterials used in the degradation of eosin Y and eosin B dyes. TEM analysis can reveal the presence of core–shell structures, lattice fringes, and crystallographic defects, providing valuable information about the material's crystallinity and homogeneity. These parameters are directly linked to photocatalytic efficiency and electron transfer processes during dye degradation.^82,91^

### 6.14 X-ray diffraction (XRD)

XRD is a powerful tool for identifying the crystalline phases and structural properties of photocatalysts. In dye degradation studies, XRD is used to confirm the successful synthesis of nanomaterials such as metal oxides (*e.g.*, TiO_2_, ZnO) or doped composites by comparing diffraction patterns with standard reference data. The technique also enables estimation of crystallite size using the Scherrer equation and provides insights into crystallinity and phase purity. For eosin Y and eosin B degradation, maintaining a stable crystal structure is essential for repeated photocatalytic performance. XRD thus plays a critical role in evaluating the structural integrity of green-synthesized photocatalysts before and after reaction cycles.^52,81,82^

### 6.15 Femtosecond transient absorption spectroscopy (fs-TAS)

While conventional characterization techniques such as UV-vis spectroscopy, FTIR, XRD, and Raman spectroscopy provide essential structural, optical, and compositional information about photocatalytic systems, they are generally insufficient to capture real-time interfacial charge transfer dynamics. To address this limitation, advanced ultrafast spectroscopic techniques, particularly femtosecond transient absorption spectroscopy (fs-TAS), are increasingly being employed to elucidate reaction kinetics at the femtosecond–picosecond scale. fs-TAS enables direct monitoring of the generation, separation, and recombination behavior of photogenerated charge carriers, offering critical insights into interfacial electron migration pathways. This is especially important for S-scheme heterojunctions, where directional charge transfer and selective recombination govern catalytic efficiency.^98^ Recent research conducted on ZnO/CdIn_2_S_4_ S-scheme heterojunctions demonstrated that fs-TA spectroscopy can directly confirm ultrafast electron migration and efficient spatial separation of photogenerated charge carriers, resulting in suppressed recombination and enhanced photocatalytic activity. The improved performance was attributed to the built-in electric field and accelerated interfacial electron transfer within the S-scheme architecture.^99^ Such mechanistic insights are highly valuable for understanding and optimizing green-synthesized photocatalysts employed in eosin dye degradation and other environmental remediation applications^99,100^

## 7. Challenges and limitations

**Fig. 5:**
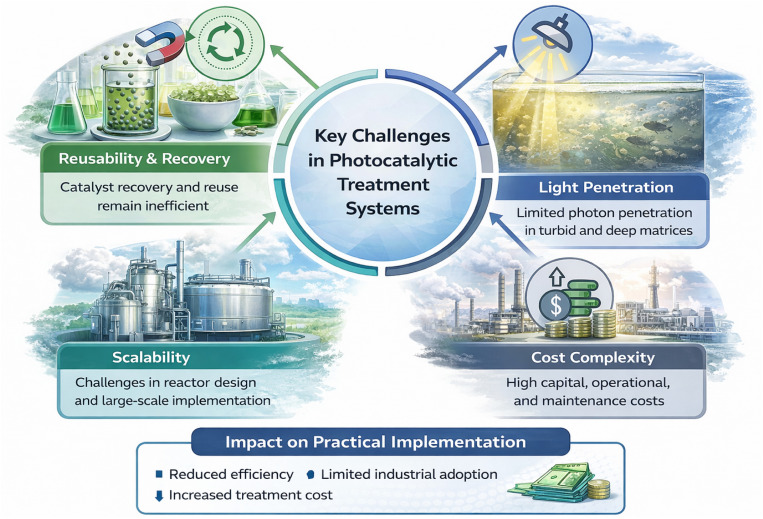
Flowchart illustrating key challenges in photocatalytic dye degradation.

Green-synthesized photocatalysts offer a sustainable route to environmental remediation, especially for dye-laden wastewater. However, despite their promising performance under controlled laboratory conditions, numerous practical, physicochemical, and engineering challenges hinder their commercial deployment. These challenges are grouped under three key categories and are elaborated in the following subsections with standard scientific context and reference to reported literature as overviewed in Fig. 5.

### 7.1 Reusability and recovery of catalysts

The reusability and ease of recovery of nanophotocatalysts are essential criteria for sustainable and economically feasible water treatment. In laboratory studies, nanomaterials are often used in suspension form, where high surface area facilitates rapid photocatalysis. However, this same feature complicates their post-treatment recovery, particularly when the catalyst is nanosized (<100 nm) and colloidally stable in solution.^40,52^

Green-synthesized catalysts from plant extracts or biowaste often retain surface organic moieties that aid dispersion and surface modification, enhancing photocatalytic activity. However, these residues may degrade under prolonged light exposure, altering catalyst stability and performance.^82^ This leads to loss of catalytic activity, particle aggregation, and photocorrosion. Over multiple cycles of reuse, the catalyst's surface may become blocked by adsorbed intermediates or may experience structural changes, reducing its effectiveness.^52^ To better understand catalyst deactivation mechanisms, surface characterization techniques such as Fourier-transform infrared spectroscopy (FTIR) and X-ray photoelectron spectroscopy (XPS) are frequently employed. FTIR can detect changes in surface functional groups and the accumulation of adsorbed organic species, while XPS provides information on surface elemental composition, oxidation states, and chemical bonding environments. Comparative analyses before and after repeated photocatalytic cycles can therefore reveal surface fouling, active-site blockage, and chemical transformations responsible for performance deterioration.^101–103^

To overcome recovery issues, magnetic photocatalysts have been developed, enabling easy retrieval *via* external magnets. However, magnetic materials themselves may suffer from oxidation, leaching, or core shell degradation after prolonged exposure to light and reactive species, necessitating surface passivation strategies such as silica coating or polymer encapsulation. Furthermore, washing, drying, and reactivation between cycles add operational complexity, highlighting the need for robust, regenerable, and immobilized catalyst systems.^104^

### 7.2 Light penetration in real effluents

One of the keys but often overlooked challenges in photocatalytic wastewater treatment, is optical accessibility, the uniform penetration of light to activate the catalyst. Unlike clear laboratory dye solutions, real effluents contain organics, colorants, particulates, and microbes that hinder light transmission and reduce efficiency.^105^

Matrix components cause the inner filter effect, where competing chromophores absorb light and limit photon flux to the catalyst. Studies show eosin dye degradation drops by \∼30% in synthetic wastewater compared to deionized water. Light scattering, biofouling, and organic matter adsorption further diminish photocatalytic efficiency in real-world systems.^106^

Advanced reactor engineering approaches such as thin-film slurry reactors, monolithic reactors, and fiber-optic illumination systems have been explored to improve light delivery. Their effective implementation requires precise alignment, high energy input, and costly hardware, limiting their deployment in low-income or decentralized treatment settings. Thus, photocatalyst development must simultaneously optimize optical activity under diffuse light and resistance to matrix interference.^107,108^

### 7.3 Scaling up from lab to industry

A major bottleneck in adopting green-synthesized nanocatalysts is scaling up, as plant extracts, microbial cultures, and biomass hydrolysates vary in composition. Such batch-to-batch inconsistency, seasonal dependence, and non-standardized methods lead to fluctuations in catalyst size, morphology, and photocatalytic performance.^109^ Ali *et al.*, reported that biogenic nanoparticles vary in size and charge with drying time, extract concentration, and pH, complicating quality control and scalability. Green synthesis typically yields only milligram-to-gram quantities in aqueous, ambient setups. Such low output limits the feasibility for large-scale wastewater treatment.^110^

Catalyst immobilization is a major challenge for continuous-flow systems, as most lab studies use suspended catalysts for maximum dye contact. Industrial use requires fixing photocatalysts on substrates like glass, membranes, polymeric supports, or meshes, which reduces active surface area and activity.^111,112^ Moreover, economic feasibility and LCA studies on green-synthesized photocatalysts remain limited; for instance, previous research showed reduced energy input using rice husk-derived catalysts, but purification and immobilization costs still hinder commercial comparability with conventional photocatalysts.^113^ In conclusion, green-synthesized photocatalysts hold clear environmental advantages but face challenges in recovery, stability, light efficiency, and scalability (Fig. 5). Bridging lab-to-field gaps demands standardized synthesis, robust immobilization, and economical reactor designs. Future efforts must unite materials science, process engineering, and policy frameworks to realize their potential in wastewater treatment.^104,110,112^

## 8. Future perspectives

Despite extensive laboratory validation of green-synthesized nanomaterials for the photocatalytic degradation of eosin dyes, several research and development directions must be prioritized to realize their practical application in wastewater treatment. Future strategies must focus on enhancing photo-efficiency, operational robustness, and environmental integration, while ensuring scalability and economic feasibility. This section presents four critical pathways supported by scientific evidence and ongoing technological advances.

### 8.1 Coupling with solar systems

The success of photocatalysis relies heavily on photon activation. While most laboratory studies employ artificial UV or visible light sources, solar energy offers a sustainable and cost-effective irradiation alternative. Many green-synthesized photocatalysts, such as TiO_2_ doped with plant-derived compounds or ZnO, possess band gaps ranging from 2.2 to 3.2 eV, making them responsive to visible sunlight.^114^

Recent studies have demonstrated that solar-irradiated photocatalytic degradation systems can reach efficiencies comparable to UV sources. Solar concentrator-integrated reactors or solar tubular photoreactors have also been proposed to increase incident light flux and reactor throughput.^115^ Furthermore, integrating photovoltaic-assisted systems with photocatalytic reactors allows for energy storage and hybrid operational modes, which are particularly useful in regions with fluctuating sunlight. Future efforts should emphasize material tuning for better solar absorption, use of broadband semiconductors, and design of solar-driven continuous-flow systems for large-scale deployment.^116^

### 8.2 Immobilized catalyst systems

Photocatalyst recovery remains a major drawback in suspended systems, but immobilization on solid supports enables reuse and reduces secondary pollution. The trade-off lies in diminished surface area and light utilization. Emerging 3D supports and transparent carriers address this by enhancing photon penetration and preserving catalytic activity.^117^ Immobilization techniques such as layer-by-layer assembly, sol–gel immobilization, electrostatic self-assembly, and electrospinning are gaining traction. However, standardization of these methods, along with durability tests under varying flow rates and light conditions, is still required. Moreover, photocatalytic membrane reactors (PMRs) combining filtration and degradation in a single step represent a growing research frontier.^118^

### 8.3 Hybrid green-chemical approaches

Although green synthesis offers environmental benefits, it often yields catalysts with modest crystallinity, broad size distribution, and low charge carrier mobility. To overcome these limitations, hybrid approaches that combine green principles with controlled doping, heterojunction engineering, and nanocomposite fabrication are emerging.^119^ Integrating green precursors into these high-performance systems enables the retention of eco-friendly synthesis benefits while achieving enhanced photocatalytic outcomes. Future developments should also explore bio-derived carbonaceous supports (graphene from biomass), MOF-based hybrids, and photo-Fenton/photo-electrocatalytic hybrid systems for dye degradation.

### 8.4 Coupled photocatalytic systems for hydrogen production

Recent advances in photocatalytic systems have shifted the research focus from conventional pollutant removal toward integrated photocatalytic conversion processes combining environmental remediation with sustainable energy generation and value-added chemical synthesis. In this regard, coupling eosin dye degradation with photocatalytic hydrogen evolution has emerged as a promising strategy for maximizing solar energy utilization and improving reaction economy.^23^ During photocatalysis, organic dye molecules can serve as sacrificial electron donors, facilitating proton reduction and enhancing H_2_ generation efficiency.^120^ Furthermore, the incorporation of spatially separated co-catalysts within heterojunction architectures enables simultaneous oxidation and reduction reactions, thereby promoting selective transformation pathways alongside wastewater purification.^121^ Such multifunctional photocatalytic systems not only enhance charge separation and redox efficiency but also expand the applicability of green-synthesized nanomaterials toward solar fuel production and fine chemical synthesis.^122^ Recent studies on S-scheme heterojunctions and co-catalyst-engineered photocatalysts have demonstrated significant improvements in hydrogen evolution activity, selective oxidation capability, and photocatalytic stability under visible-light irradiation.^23^ In addition to hydrogen evolution, future photocatalytic systems may integrate eosin dye degradation with photocatalytic CO_2_ reduction, enabling simultaneous wastewater remediation and carbon valorization. In such systems, photogenerated holes participate in the oxidation and mineralization of dye molecules, while photogenerated electrons drive the reduction of CO_2_ into value-added products such as methanol, methane, carbon monoxide, or formic acid. This dual-functional approach can improve charge-carrier utilization efficiency, suppress electron–hole recombination, and simultaneously address water pollution and greenhouse gas emissions.^123,124^ Consequently, coupling dye degradation with CO_2_ photoreduction represents a promising research direction for next-generation sustainable photocatalytic technologies.

### 8.5 Coupled piezo-photocatalytic systems for enhanced performance

Recently, coupled piezo-photocatalytic systems have emerged as a promising strategy for enhancing photocatalytic efficiency by addressing key limitations such as rapid charge carrier recombination and limited mass transfer.^125^ In these systems, mechanical energy derived from external stimuli such as water flow, vibration, or ultrasonic waves is converted into localized electric fields *via* piezoelectric materials. This process facilitates efficient separation and migration of photogenerated electron–hole pairs, thereby improving reactive oxygen species generation and interfacial charge-transfer kinetics.^126^ The synergistic interaction between piezoelectric and photocatalytic effects further enhances light utilization and accelerates surface redox reactions, making these systems highly promising for advanced environmental remediation and sustainable energy applications^125,127^

### 8.6 Need for pilot-scale demonstrations

Despite growing interest in green photocatalysts, there remains a paucity of pilot-scale demonstrations. Most existing studies focus on synthetic aqueous dye solutions under ideal lab conditions. However, real wastewater is complex, containing multiple pollutants, turbidity, variable pH, and competing absorption species. Scaling photocatalysis from lab to field requires demonstration units capable of operating under natural solar light, variable flows, and realistic retention times. Challenges include large-scale catalyst production, maintaining activity, immobilization costs, and post-treatment effluent handling. Life-cycle assessment (LCA) and techno-economic analysis (TEA), though vital for policy and investment, are often overlooked. Standard protocols, field validation, and academia–industry partnerships are essential for real-world adoption.^128,129^

## 9. Conclusions

This review highlights the urgent need for sustainable approaches to address eosin dye contamination in industrial wastewater, with green-synthesized nanomaterials emerging as promising photocatalysts. Plant-, microbial-, and biowaste-derived nanocatalysts show strong degradation potential under visible or solar light, supported by established photocatalytic mechanisms and kinetic models. The importance of advanced characterization and monitoring methods for evaluating degradation efficiency, intermediates, and catalyst stability is emphasized. Despite encouraging laboratory outcomes, challenges remain in reusability, light penetration, and scale-up. Future directions point toward solar-driven, immobilized, and hybrid green systems validated at pilot-scale for real-world application. This review demonstrates that the remediation of eosin Y and eosin B dyes, persistent, toxic, and increasingly prevalent pollutants in industrial wastewater, requires innovative and sustainable treatment solutions beyond conventional methods. Green-synthesized nanomaterials have emerged as highly promising photocatalysts, offering efficient degradation under visible and solar light while reducing environmental and economic burdens associated with traditional catalyst fabrication.

Biogenic photocatalysts derived from plant extracts, microorganisms, and biowaste exhibit excellent optical properties, tunable surface chemistry, and strong photocatalytic activity linked to well-established degradation pathways and kinetic models. Their performance is further elucidated using advanced structural and analytical tools, enabling deeper understanding of catalyst stability, active sites, electron–hole dynamics, and mineralization of degradation intermediates. Although laboratory-scale results are compelling, practical implementation remains constrained by challenges such as catalyst recovery, limited operational stability, mass-transfer limitations, and insufficient light penetration in real wastewater matrices. Bridging this gap requires strategic efforts to design immobilized nanocatalysts, solar-driven reactors, hybrid photocatalytic–biological systems, and scalable engineering approaches that maintain efficiency under industrial conditions. Overall, biogenic nanomaterials represent a transformative pathway toward greener and more efficient photocatalytic degradation of eosin dyes. Advancing pilot-scale demonstrations, optimizing catalyst durability, and integrating renewable energy inputs will be critical to translating laboratory advances into reliable, eco-friendly wastewater treatment technologies capable of meeting global sustainability goals.

## Author contributions

Komal Shah: conceptualization, writing – original draft. Muhammad Zubair: supervision, writing – review & editing, final approval. Rida Nisar: data curation, literature review. Sobia Kunbhar and Gull Sabah Mirza: investigation, writing – review & editing. Mustafa Tuzen: supervision and visualization. A. Shah: investigation, visualization, writing – review & editing.

## Conflicts of interest

The authors declare that they have no known competing financial interests or personal relationships that could have appeared to influence the work reported in this paper.

## Data Availability

Not applicable. No new data were generated or analyzed.
